# A Comprehensive Transcriptomic and Proteomics Analysis of Candidate Secretory Proteins in Rose Grain Aphid, *Metopolophium dirhodum* (Walker)

**DOI:** 10.3390/cimb46120798

**Published:** 2024-11-23

**Authors:** Atsbha Gebreslasie Gebrekidan, Yong Zhang, Julian Chen

**Affiliations:** State Key Laboratory for Biology of Plant Diseases and Insect Pests, Institute of Plant Protection, Chinese Academy of Agricultural Sciences, Beijing 100875, China; atsbha1415@gmail.com

**Keywords:** unigene, functional annotation, salivary protein, sequence similarity

## Abstract

The Rose grain aphid, a notable agricultural pest, releases saliva while feeding. Yet, there is a need for a comprehensive understanding of the specific identity and role of secretory proteins released during probing and feeding. Therefore, a combined transcriptomic and proteomic approach was employed in this study to identify putative secretory proteins. The transcriptomic sequencing result led to the assembly of 18,030 unigenes out of 31,344 transcripts. Among these, 705 potential secretory proteins were predicted and functionally annotated against publicly accessible protein databases. Notably, a substantial proportion of secretory genes (71.5%, 69.08%, and 60.85%) were predicted to encode known proteins in Nr, Pfam, and Swiss-Prot databases, respectively. Conversely, 27.37% and 0.99% of gene transcripts were predicted to encode known proteins with unspecified functions in the Nr and Swiss-Prot databases, respectively. Meanwhile, the proteomic analysis result identified, 15 salivary proteins. Interestingly, most salivary proteins (i.e., 60% of the proteins) showed close similarity to *A. craccivora*, while 46.67% showed close similarity to *A. glycines*, *M. sacchari* and *S. flava*. However, to verify the expression of these secretory genes and characterize the biological function of salivary proteins further investigation should be geared towards gene expression and functional analysis.

## 1. Introduction

Aphids (Hemiptera: Aphididae) are voraciously phytophagous insects that ingest plant phloem sap via their needle-like stylets (mouth parts). They are unusual herbivores because their feeding site is a single phloem cell in the sieve element buried deep within plant tissues; yet according to Gupta [1,2], they represent one of the most important insect pests in temperate regions. Worldwide, there are about 5558 species of aphids, which are classified into 703 genera and 30 subfamilies [3]. Among recorded aphid species, around 450 of them feed on crop plants, and 250 aphid species are considered economically important agricultural pests causing significant economic losses [4,5]. Four aphid species cause serious damage to wheat farms in China. The major aphid species infesting wheat in China are the grain aphid (*Sitobion avenae* Fabricius), the green bug (*Schizaphis graminum* Rondani), the bird cherry oat aphid (*Rhopalosiphum padi* Linnaeus) and the rose grain aphid (*Metopolophium dirhodum* Walker) [6]. Some Phloem feeding insects, such as pea aphids (*Acyrthosiphon pisum* Harris) have a wide host range often colonizing many leguminous plant species. Unlike the other types of aphids, *M. dirhodum* are primarily restricted to crops that belong to the Rosaceae (mainly cherries and peaches), but are a secondary pest of cereal crops (wheat and other cereal crops).

Certain plants have the ability to withstand aphid feeding without negative consequences, either through inherent genetic resistance or by adjusting their interactions with aphids. This natural defense mechanism has proven effective in controlling aphids in various crops. The interactions between plants and aphids can be seen as a process of coevolution, where both parties adapt to each other [7]. Aphids extract nutrients from host plants by piercing the phloem to access sap with their stylets, releasing saliva from salivary glands that contain effector proteins to counter plant defenses [8,9]. The composition of watery saliva, containing a diverse blend of enzymes and other substances, varies significantly among aphid species and even within the same species depending on their diet [10,11,12]. The ability of aphids to adapt to different host plants is closely tied to the diversity in watery saliva content. This generally involves the secretion of secretory molecules, specifically effectors that target host molecules [13]. Many studies have shown that insects, including aphids, produce and secrete effectors that suppress or induce plant defense responses [14,15,16,17]. These aphid secretory proteins are thought to be produced predominantly inside the head and are secreted along with saliva during probing and feeding [18,19,20]. Nonetheless, limited research has delved into the connection between feeding patterns and the genes expressed in aphid salivary glands during shifts in host preferences.

The recent availability of data on the genome and transcriptome sequences of aphids has enabled the development of an approach that can identify potential secretory proteins from aphids [8,14,18]. Bioinformatics pipelines used to identify putative secreted proteins have been developed and applied to several aphid species [16,21]. In addition, saliva collection methods based on artificial feeding systems combined with mass spectrometry allow for the detection of many proteins in the saliva of several types of aphids released during probing and feeding [14,22]. Significant progress has been made in the identification and functional annotation of potential effectors of economically important aphid species for cereals, such as bird cherry-oat aphids, and green bug aphids, through proteomic and transcriptomic analyses. However, limited gene and/or proteomic data for rose-grain aphids have been documented, despite the fact that rose-grain aphids are the fourth most dominant and destructive aphids affecting wheat production in many temperate regions, including China. Furthermore, the molecular mechanisms underlying these differences have not been documented because there is a lack of sufficient genetic data for rose-grain aphids in public databases. Consequently, this study employed a combined transcriptomic and proteomic approach to mark an initial step towards identifying candidate secretory proteins involved in plant infestation and environmental adaptation.

## 2. Materials and Methods

### 2.1. Aphid Handling and RNA Preparation

Both apterous and alate adult rose-grain aphids from a clonal lineage were reared on susceptible wheat (*Triticum aestivum* L. Var. Zhongmai-175) in a controlled laboratory setting. Aphid heads along with salivary glands were dissected under a stereomicroscope and temporarily stored in liquid nitrogen. RNA was extracted using TRIzol reagent and then purified using a Tianmobio total RNA extraction kit. The concentration of total RNA was measured with the aid of a Nano-drop spectrophotometer (IMPLEN, CA, USA). The quantity and integrity of the RNA was assessed using the RNA Nano 6000 Assay Kit (Agilent Technologies, Santa Clara, CA, USA).

### 2.2. cDNA Library Preparation, Cluster Generation and Sequencing

The cDNA library construction process began with using 1.5 µg of RNA sample as the input material. The synthesis of the cDNA library involved fragmenting mRNA using divalent cations in the presence of random hexamer primers and M-MuLV Reverse transcriptase. DNA Polymerase I and RNase H were employed to construct the first and second cDNA strands. The overhangs were then converted into blunt ends through exonuclease/polymerase activity. Three microliters of enzyme were used with size-selected, adaptor-ligated cDNA at 37 °C for 15 min, followed by 5 min at 95 °C. PCR amplification was carried out using Phusion High-Fidelity DNA polymerase, universal PCR primers, and Index Primer. The PCR products were purified using the AMPure XP system (Beckman Coulter, Beverly, MA, USA), the library quality and insert size was assessed with the aid of the Agilent Bioanalyzer 2100 system (Agilent Technologies, CA, USA). Furthermore, the library’s quantity was measured using a Qubit 2.0 Fluorometer. A paired-end library of 250–300 bp size was constructed according to the user’s instructions (Illumina’s protocol). Subsequently, the samples were clustered with the aid of cBot Cluster Generation System along with the TruSeq PE Cluster Kit v3-cBot-HS from Illumina (NEB, Ipswich, MA, USA).

### 2.3. Quality Control, Transcriptome Assembly, and Gene Annotation

The first stage of the analysis involved the processing of raw data (raw reads) into the FASTQ format through custom Perl scripts. Subsequently, clean reads were acquired by removing intact adapter reads (the proportion of reads with connectors or linkers), ambiguous reads (the proportion of reads with unspecified base information), and low-quality reads (Q Phred ≤ 20 is the number of bases in the total read length of 50% or more of the reads) from the raw data. At the same time, the clean data was evaluated for Q20 (the percentage of bases with a Q_Phred_ value greater than 20 to the total bases, where Q_Phred_ = −10log10(e)), Q30 (the percentage of bases with a Q_Phred_ value greater than 30 to the total bases), GC content, and sequence duplication level. The transcriptome was assembled using Trinity software [23] with min_kmer_cov set to 2, and all other parameters with default settings. Following the assembly, reads were aligned back to the transcriptome assembly using the align_and_estimate_abundance.pl script, with RNA-seq via the Expectation Maximization (RSEM) method and Bowtie to estimate abundance and map of the reads. Transcripts with fewer than 0.5 transcripts per million mapped reads (TPM) or representing less than 10% of the expression value of the dominant isoform for each unigene were excluded from the transcriptome assembly. Protein coding regions were identified using Trans-decoder v.3.0.1, retaining the highest scoring open reading frame (ORF) for each transcript with the single_best_orf option. Finally, transcripts without open reading frames were removed from the assembly.

A functional annotation of the derived genes was done using the Basic Local Alignment Search Tool (BLAST2go) against public databases, including the National Center for Biotechnology Information (NCBI), Non-redundant Nucleotide Acid Database, Non-redundant Protein Database, and BLASTx against the Swiss-Prot Database. The best matched alignment results were considered to annotate all unigenes; if the alignment results among these databases differed, preference was given to the results from the NR database. All genes that matched the NR database entries were further classified according to Gene Ontology terms (matched genes with an e-value ≤ 10^−6^), Eukaryotic Orthologous Groups (KOG)/Clusters of Orthologous Groups of proteins (COG) (matched genes with an e-value ≤ 10^−3^), and Kyoto Encyclopedia of Genes and Genomes (KEGG). Automatic Annotation Server (KAAS) was used with a cut-off e-value ≤ 1.0 × 10^−1^. Gene homology was established by searching public databases, such as non-redundant proteins (Nr, with genes having an e-value ≤ 10^−5^), nucleotide sequences (Nt, with genes having an e-value ≤ 10^−5^), Pfam (with genes having an e-value ≤ 0.01), and Swiss-Prot (with genes having an e-value ≤ 1.0 × 10^−5^), for all genes. Gene Ontology enrichment analysis of assembled unigenes was conducted using the GOseq R package (1.10.1). The KOBAS software [24] was employed to assess the statistical enrichment of all unigenes in the KEGG pathways.

### 2.4. Prediction of Secretory Proteins

The coding sequences of every unigene were translated into peptide sequences and subsequently analyzed using a web-based server to assess the presence secretion signals (signal peptides) associated with the N-terminal of amino acid sequence (https://services.healthtech.dtu.dk/services/SignalP-6.0/, accessed on 2 February 2024). Those sequences with signal peptides were also subjected to transmembrane domain prediction web-based tool (https://dtu.biolib.com/DeepTMHMM, accessed on 5 February 2024) to investigate the presence of transmembrane domains. Ultimately, the cluster of protein sequences which had signal peptides and those devoid of transmembrane domains were reported as candidate secretory proteins.

### 2.5. Saliva Collection, Extraction and Protein Identification

#### 2.5.1. Saliva Collection and Extraction

**Figure 1:**
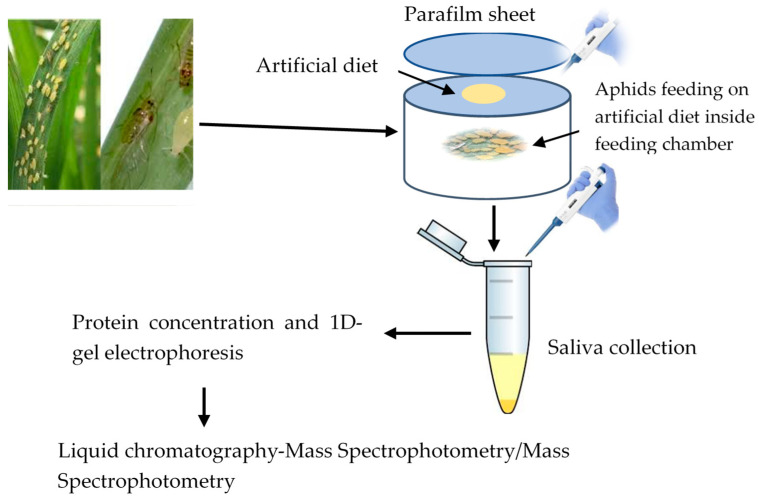
Aphid feeding on artificial diet and saliva collection.

The young rose grain aphids are born as fully developed nymphs and start feeding on plant saps immediately after born. Thus, both adult and immature rose grain aphids (third and fourth nymphal instars) were collected from wheat plants and placed inside the hole of polyurethane tubes. The tube comprised of chemically defined sterile diets; 15% sucrose, 100 mM L-serine, 100 mM L-methionine, and 100 mM L-aspartic acid, with a pH of 7.2 adjusted using potassium hydroxide (KOH) [20]. The diet was prepared under aseptic conditions and filtered using a 0.22 μm syringe filter (Millipore, MA, USA). Approximately 60,000 aphids were confined in a total of 240 polyurethane tubes (250 aphids in each tube), with 1 mL of the diet provided between two layers of parafilm membranes stretched over one end of the tube (Neenah, WI, USA) (Figure 1). Saliva collection was performed 24 h after feeding and eventually pooled into four collection tubes (50 mL each).

**Figure 2:**
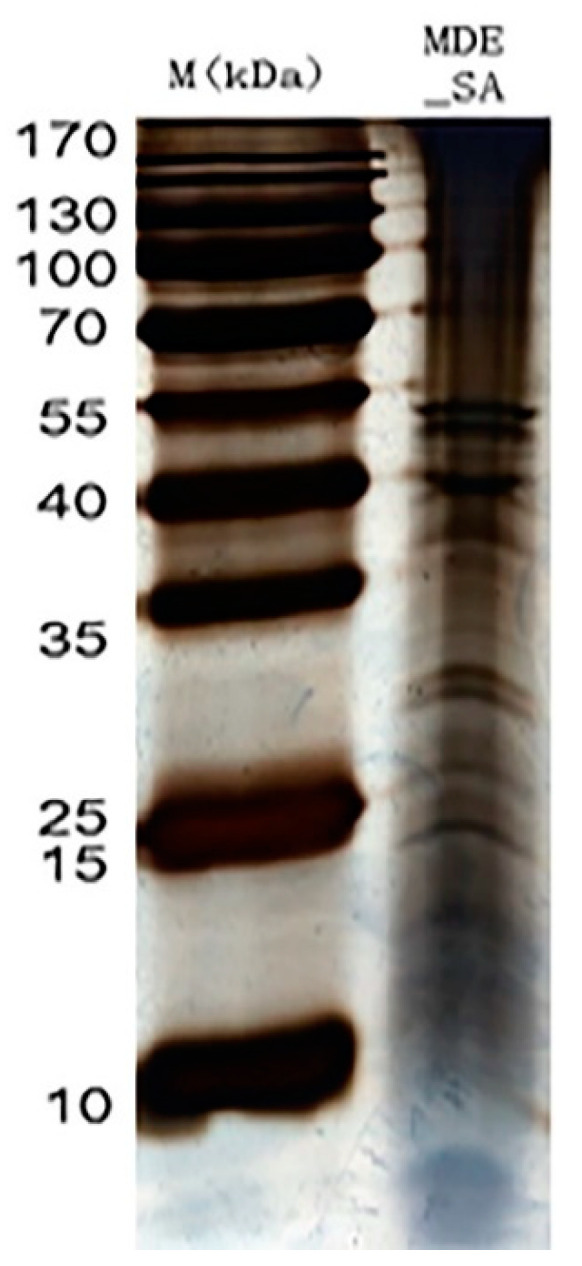
The schematic illustration of salivary protein bands of *M. dirhodum* (MDE_SA) in reference to marker (M).

Ultrafiltration was performed with a 3-kDa molecular-weight cut-off Amicon Ultra-4 Centrifugal Filter Device (Millipore, Bedford, MA, USA) at 5000× *g* at 4 °C for 30 min. The concentrated samples were then precipitated using a trichloroacetic acid protein precipitation kit (Sangon, Shanghai, China). The pellets were solubilized in 200 µL of SDT buffer and Bicinchoninic Acid (BCA) protein determination assay (Bio-Rad, Hercules, CA, USA). The proteins were then separated on a gel using one dimensional SDS-PAGE (1-DE) and stained with Coomassie Blue R-250 dye to visualize protein bands. Protein bands that were visible were carefully sliced using sterile blades followed by the addition of 100 mL of a 100 mM ammonium bicarbonate (at a 1:1 ratio by volume) and 500 mL of pure acetonitrile until the Coomassie Blue R-250 dye was completely removed. An amount of 100 µL of trypsin buffer was added to the gel pieces until the gel was completely saturated inside fridge. The tubes containing gel pieces were placed in an air-circulating thermostat, where they underwent overnight digestion using sequencing-grade modified porcine trypsin (13 ng µL^−1^, Promega, Madison, WI, USA) in a 50 mM ammonium bicarbonate solution. The peptides were then extracted from the supernatant using a solution of 30% acetonitrile/0.2% trifluoroacetic acid, followed by 60% acetonitrile/0.2% trifluoroacetic acid. The resulting peptides were dehydrated under a vacuum and reconstituted in 0.1% formic acid for downstream analysis. The saliva sample consistently showed bands at 15, 25, 40 and, 55, and between 25 and 35 kDa (Figure 2). Occasional blurred bands observed in the gel were due to sample degradation and were not further examined. Only the consistently appearing bands on the 1-DE gels were cut out and analyzed using LC-MS/MS.

#### 2.5.2. Liquid Chromatography Tandem Mass Spectrometry (LC-MS/MS)

The SDS-PAGE fractionated proteins were subjected to LC-MS/MS analysis performed on a Q Exactive mass spectrometer (Thermo Fisher Scientific, Waltham, MA, USA) coupled with Easy nano-liquid chromatography (Thermo Fisher Scientific). While the sample eluted from the LC column, thousands of mass spectra were acquired, and subsequently, the mass spectrum of all peptides was measured at that time point. The mass analyzer separated the peptides based on their mass-to-charge ratio, and the detector detected precursor peptide ions. The most abundant precursor peptide ion (300–1800 *m*/*z*) was further fragmented by collision with neutral gas molecules after passing through the filter unit of the mass spectrometer.

#### 2.5.3. Proteomic Data Analysis and Similarity Search

The out-puts of mass spectrometry (MS) were analyzed using the MaxQuant and MASCOT search engines to look for MS/MS spectra against datasets from the Pea Aphid Genome Sequencing Project (Matrix Science, London, UK; version 2.4). The MS data were also searched against the UniProtKB database. The search followed an enzymatic cleavage rule for trypsin, and allowed a maximum of two missed cleavage sites and a peptide mass tolerance of 20ppm for fragment ions. The cut-off global false discovery rate (FDR) for peptide and protein identification was set to 0.01 or (FDR ≤ 0.01).

BLAST score ratio (BSR) tests were employed to compare the proteome similarities among different aphid species, including *M. dirhodum*, *Aphis craccivora*, *Aphis glycines*, *Acyrthosiphon pisum*, *Aphis gossypii*, *Schizaphis graminum*, *Sitobion avenae*, *Rhopalosiphum padi*, *Myzus persicae*, *Melanaphis sacchari*, and the yellow sugar cane aphid, *Sipha flava*. The sequence comparisons were conducted using the BLAST search available at http://blast.ncbi.nlm.nih.gov/Blast.cgi, accessed on 5 February 2024. The BSR index was determined by dividing the BLAST query score by the reference score and normalizing the result to a scale from 0 to 1. The reference library included *A. craccivora*, *A. glycines*, *A. pisum*, *Aphis gossypii*, *Schizaphis graminum*, *S. avenae*, *R. padi*, *M. persicae*, *M. sacchari*, and *S. flava*.

$$BLAST\;score\;ratio\;1\;(BSR1)\;=\;\frac{Query\;sequence}{Reference\;sequence\;1}$$

## 3. Results

**Figure 3:**
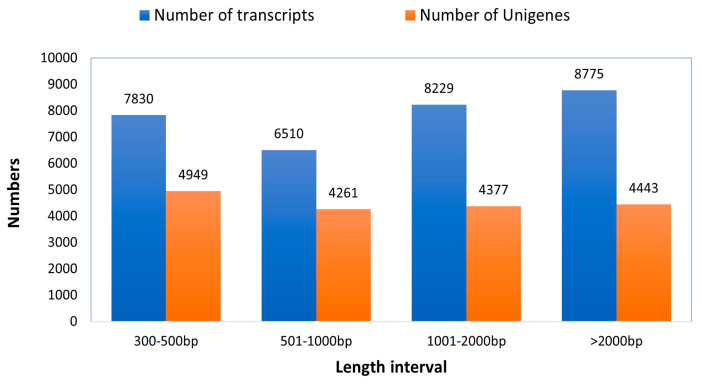
Sequence length distribution of genes and transcripts of the trinity generated with de novo assembly driven out of the raw reads of rose grain aphid transcriptome.

Altogether, 47,565,328 bp of raw reads were retrieved from the sequencing of the established library. After removing low-quality reads, ambiguous reads, and those related to adapters, 46,238,772 clean reads were produced using Illumina paired-end RNA-seq technology, achieving a Q20 value of 97.61% in the transcriptomic assembly. Approximately 39,293,370 reads (84.98% of the total clean reads) were successfully aligned to the reference sequence (the longest transcript unigene). After filtering out adapter-related reads (609272), ambiguous reads (18194), and low-quality reads (35812), clean reads were retained. The transcriptome of the rose-grain aphid head was then *de novo* assembled using the short-read assembling program (Trinity), resulted in the clustering of 31,344 transcripts and 18,030 genes (Table 1). The transcripts varied in length from 301 to over 2000 base pairs, with an average size of 1532 base pairs. Out of the transcripts, 7830 (24.98%) were in the range of 301 to 500 base pairs, 6510 (20.77%) were between 501 to 1000 base pairs, 8229 (26.25%) ranged from 1001 to 2000 base pairs, and 8775 (28%) were longer than 2000 base pairs. Furthermore, among the genes that were assembled, 4949 (27.45%) had ranged between 301 to 500 base pairs, and 4261 (23.63%) fell within the range of 501 to 1000 base pairs. Among the genes analyzed, 4377 (24.27%) fell within the range of 1001 to 2000 base pairs, while 4443 (24.64%) surpassed the 2000 base pair mark (Figure 3). The mean size of the genes derived from the transcripts was 1413 base pairs. In comparison, the transcripts produced by Trinity had an average length of 1532 base pairs (N50 = 2335 bp), demonstrating longer transcripts than the average gene length of 1413 base pairs (N50 = 2205 bp). All unigenes assembled in this study are illustrated in Supplementary Table S1. The raw sequencing data have been submitted to the Sequence Read Archive (SRA) of the National Center for Biotechnology Information (NCBI) database with the accession number PRJNA1134911 (http://www.ncbi.nlm.nih.gov/bioproject/PRJNA1134911, accessed on 5 February 2024).

### 3.1. Functional Annotation of Gene Transcripts

Head tissue libraries were annotated utilizing BLASTx against the NCBI database, and all unigenes underwent homology searches. The functional annotation of all unigenes from *M. dirhodum* were subjected to a BLAST search against seven databases including NCBI’s non-redundant protein (Nr) and nucleotide (Nt) sequences, protein family (Pfam), the manually annotated and reviewed Swiss-Prot protein sequence database, Eukaryotic Clusters of Orthologous Groups of proteins (KOG/COG), the Kyoto Encyclopedia of Genes and Genomes (KEGG) Ortholog database and Gene Ontology (GO). This comprehensive approach was employed to annotate the functions of each gene transcript. Of the total assembled unigenes (18,030), 12,589 (69.82%), 14,253 (79.05%), 5587 (30.98%), 9467 (52.5%), 9314 (51.65%), 9314 (51.65%) and 5850 (32.44%) genes were matched to known proteins in the non-redundant protein sequences, non-redundant nucleotide sequences (Nt), KEGG Orthology (KO), Swiss-Prot, Protein family (Pfam), Gene Ontology (GO), and Eukaryotic Orthologous Groups (KOG) databases, respectively (Table 2). The alignment result of the total unigenes were not similar across all databases and hence, the NR database results were preferentially employed to annotate all unigenes.

**Figure 4:**
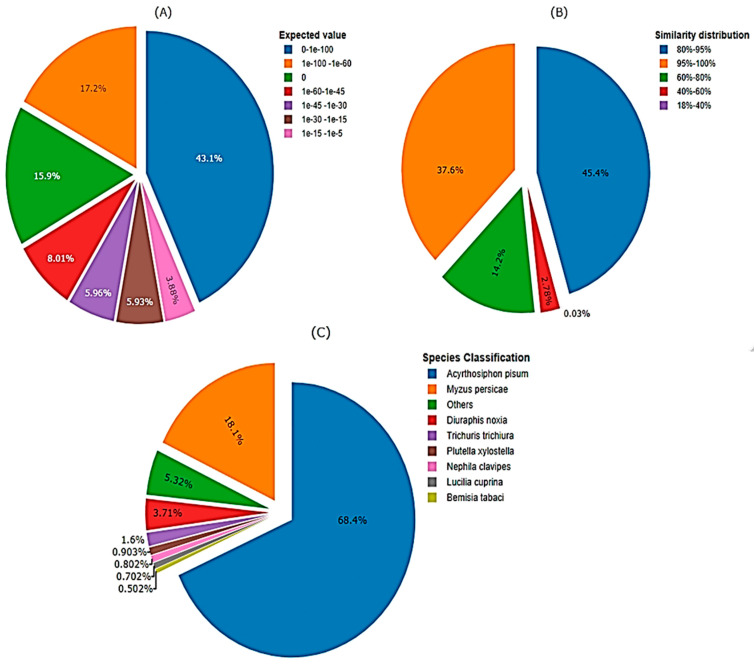
Homology analysis of *M. dirhodum* genes against non-redundant protein sequence database. (**A**) Expected threshold value (E-value) in which the number BLAST hits of each gene appear by chance (e-value < 1.0 × 10^−5^), (**B**) Similarity distribution, (**C**) Species classification based on sequence similarity of genes among different organisms.

The newly de novo assembled transcriptomic sequences, were examined using a BLAST search against the NCBI database to deduce the functional and structural characteristics of the proteins encoded by these transcripts. The comparison involved matching all coding sequences from *M. dirhodum* genes with established transcriptomic sequences from various aphid species found in publicly accessible protein databases, such as Nr, Swiss-Prot, and Pfam. Consequently, the de novo assembly of *M. dirodum* transcripts resulted in the prediction of 12,589, 9467 and 9314 functionally characterized and hypothetical gene products (proteins) in the Nr, Swiss-Prot and Pfam genome databases, respectively. The e-value distribution of the top matches against the nr database revealed that, 17.18% of the sequences exhibited strong homology (e-value < 1.0 × 10^−60^), with most e-values falling between 0 and 1.0 × 10^−100^ (Figure 4A). Furthermore, the similarity distribution indicated that 97.18% of the unique sequences with top matches had a similarity greater than 60%, while 2.82% of the hits showed a similarity below 60% (Figure 4B). Additionally, the sequences of assembled unigenes were compared to genome sequences from other organisms available in major databases to check for conserved genes through a homologous sequence search. The majority of unigene sequences (68.2%) were matched with *A. pisum* sequences, followed by *M. persicae* (18%) (Figure 4C). However, a portion of the gene transcripts (13.7%) showed less similarity to other aphid species.

### 3.2. Gene Ontology and Eukaryotic Orthologous Groups Classification (KOG)

**Figure 5:**
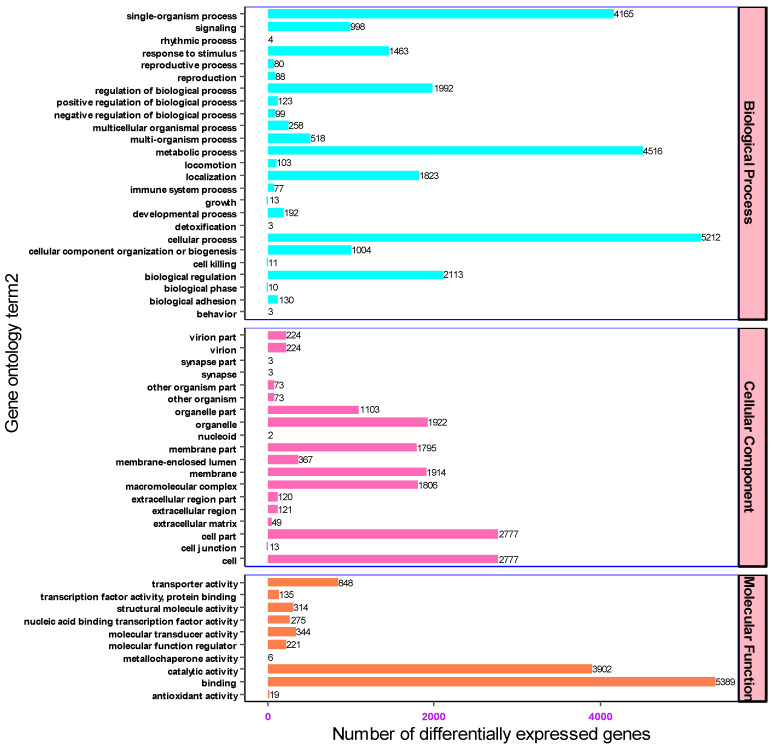
The biological, molecular and cellular classification of genes associated with each functional category.

The gene transcripts characterized based on the Nr and Swiss-Prot databases were further annotated against gene ontology to determine the number of genes associated with each functional category. Of all the assembled unigenes (18,030), 9314 were assigned to three functional groups. The three most abundant functional categories were biological processes, cellular components, and molecular function with 4493 (48.24%), 2762 (29.65%) and 2059 (22.1%) unigenes associated with each functional category, respectively. Accordingly, a wide distribution and assignment of unigenes were implicated in biological process. Based on the GO enrichment analysis results, the top 54 over-represented genes were cellular processes (5212), metabolic processes (4516), single organism processes (4165), biological regulation (2113), regulation of biological process (1992) and localization (1823) respectively. On top of that among the genes associated with molecular functions, 5389, 3902, and 848 unigenes were involved in binding, catalytic and transport activities respectively. In light of cellular components, most unigenes were associated in cells (2777), cell parts (2777), organelles (1922) and membranes (1914) (Figure 5).

**Figure 6:**
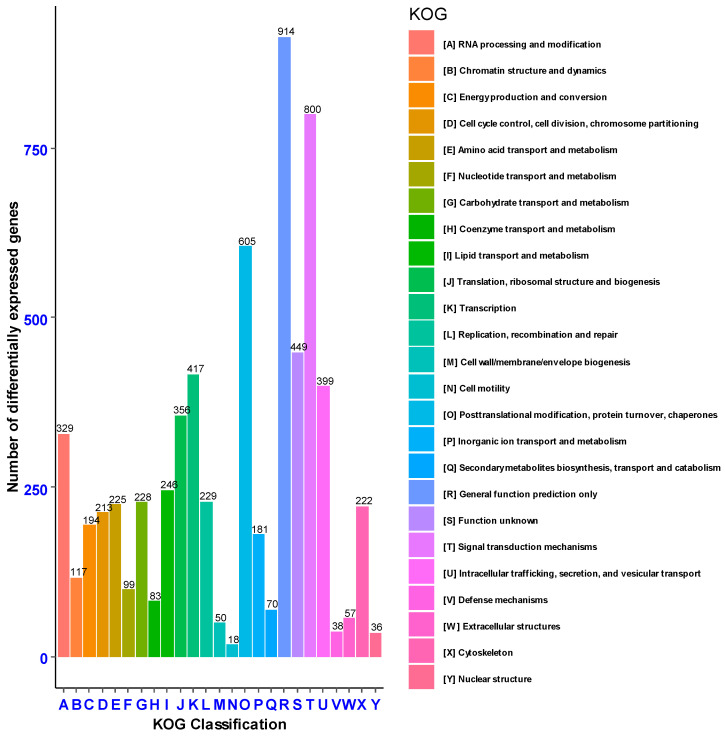
Genes annotated based on Eukaryotic Orthologous Groups classification (KOG).

Eukaryotic Orthologous Groups (KOG) were extrapolated from Clusters of Orthologous Groups (COG) to specifically predict the sequence of unknown genes from previously identified and stored in known databases of evolutionarily related sequences of different organisms. Based on KOG, 6575 genes were annotated into known (6126 genes) and unknown (449 genes) functional ortholog groups. Among the known functional groups, the highest percentage of genes (914, 13.9%) was annotated to the general function prediction, followed by signal transduction mechanisms (800, 12.17%), post-translational modifications, protein turnover and chaperones (605, 9.2%) (Figure 6). 

### 3.3. Metabolic Pathway Analysis by Kyoto Encyclopedia of Genes and Genomes (KEGG)

The Kyoto Encyclopedia of Genes and Genomes (KEGG) pathway was used to describe the structural elements of the gene products and metabolic pathways in the cell. Based on KEGG, 5587 gene transcripts were assembled from the transcriptomic sequences of the heads of *M. dirhodum* aphids. The transcripts of these genes were annotated to 228 functional pathways. 

**Figure 7:**
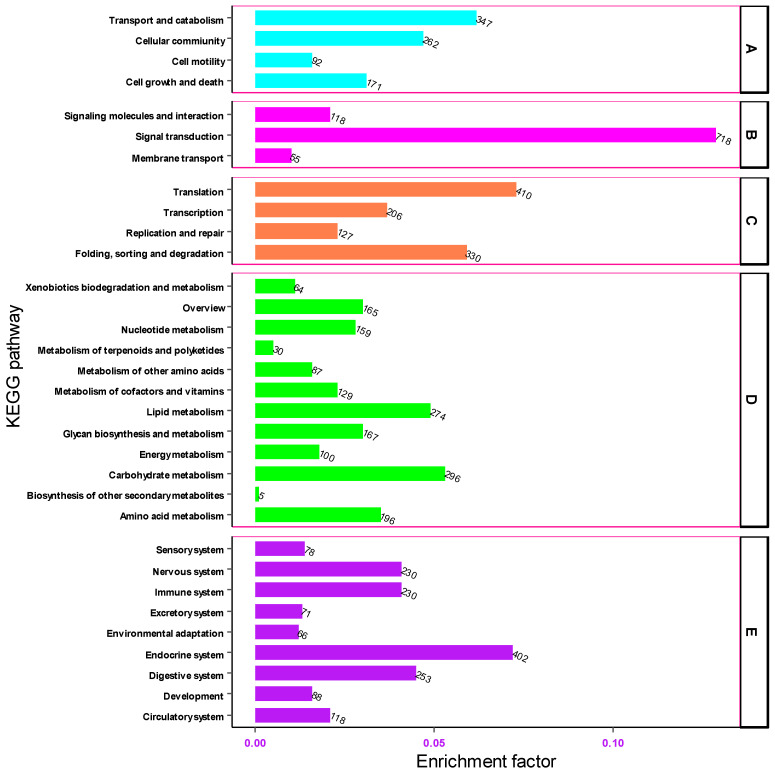
Functional classification of unigenes associated with cellular processes (A), environmental information processing (B), genetic information processing (C), metabolism (D) and organismal systems (E) pathways.

Based on KEGG pathway annotations, the transcripts of genes associated with the KEGG pathway were grouped into five functional categories: Cellular Processes, Environmental Information Processing, Genetic Information Processing, Metabolism and Organismal Systems. In the cellular process category, the highest number of unigenes were associated with the transport and catabolism (347, 5.74%) and cellular (262, 4.33%) functional pathways, respectively. In the environmental information processing category, a significant number of unigenes were implicated in signal transduction (718, 11.88%) while extremely few numbers of genes were involved in membrane transport (55), signal molecules and interaction (118). Of these genes associated with the genetic information processing category, the highest number of genes (410, 6.78%) were involved in translation and folding, followed by sorting, and degradation (330, 5.46%) KEGG pathways. In the metabolism group, the carbohydrate metabolism pathway was significantly enriched with 296 (4.89%) unigenes. In the organismal systems category, the number of the transcripts of genes associated with the endocrine system, digestive system, immune system and nervous system were 402 (6.65%) and 253 (4.18%), 230 (3.8%) and 230 (3.8%), respectively (Figure 7). These findings offer significant insight into the environmental information processing pathways within the head tissue of *M. dirhodum*. They may serve as a foundation for future research aimed at identifying and characterizing the genes and transcripts involved in key signal transduction pathways.

### 3.4. Putative Secretory Proteins

Salivary proteins are believed to be involved in plant interactions only when they are secreted during aphid probing and feeding. All genes were compared with the priority order of non-redundant protein sequences (NR protein), protein family (Pfam) and the Swiss-Prot protein library to analyze the presence of signal peptides. Therefore, in this study, 705 putative secretory genes were predicted of the total unigenes assembled from the transcriptomic sequence of head tissue. These proteins had signal peptides associated with the N-terminal of the amino-acid sequence along with one and /or zero transmembrane domains, indicating their secretory nature (Supplementary Table S2). Of all clusters of transcripts for secretory genes, 504 (71.5%), 487 (69.08%) and 429 (60.85%) genes were characterized as encoding known secretory proteins with known functions in the Nr, Pfam and Swiss-Prot databases, respectively. However, 193 (27.37%) and 7 (0.99%) transcripts of genes were predicted to encode known proteins with uncharacterized functions in the Nr and Swiss-Prot databases, respectively. In contrast, the type and function of gene transcripts of some secretory genes were not known in the NR (8 gene), Swiss-Prot (269 genes) and Pfam (218 genes) databases. Most functionally annotated products of putative secretory genes were highly conserved (orthologous) among different aphid species, including pea aphids. Thus, they had similar biological and molecular functions in plant-aphid interactions during feeding. 

### 3.5. Salivary Proteins and Sequence Similarity Among Aphid Species

The release of aphid salivary proteins plays an indispensable role during aphid-plant interaction. A thorough examination of saliva from *M. dirhodum* uncovered the existence of 37 proteins (Supplementary Files One & Two). However, the majority of those proteins, 57% (or 22 proteins), had single peptides and were thus rejected as they typically represent false positives. Of all the detected proteins, only 14 had two or more peptides with multiple spectra, with the exception of one with single peptide that had multiple spectra (Table 3). Most of these salivary proteins, 73.3% (or 11 proteins), lacked secretion signals (signal peptides) at the N-terminal of the amino acid sequence. Among the salivary proteins, only 26.67% (or 4 proteins), specifically Glucose dehydrogenase-like protein 2, Heat shock protein 70, Putative sheath protein, and Vitellogenin domain-containing protein, had signal peptides associated with their protein sequence. The identification of salivary proteins like glucose dehydrogenase, putative sheath, heat shock protein, peroxiredoxin, and Vitellogenin proteins in the saliva of *M. dirhodum*, holds the practical significance of secretory proteins that influence the range of host plant exploitation and determine the ability of this aphid to adapt to different crop varieties. Not all proteins identified through MS analysis of the collected saliva showed secretion signals. Thus, there may be alternative mechanisms for protein transport into saliva that should also be considered. As a result, it is probable that our assumptions have led to the exclusion of some salivary proteins from our predicted pool of candidate secretory proteins. It is not always true that all salivary proteins are expressed in the head tissue of sap sucking insects. As hemolymph is continually circulated through the salivary glands, it is likely that various biological molecules, including proteins originating from different tissues, are transported into the salivary glands along with the hemolymph or through other unknown mechanisms. 

The identity of the majority of the salivary proteins were determined based on a sequence homology search (38.8%) followed by transcript expression levels (13.3%) and sequence prediction (20%). Among the salivary proteins actin had depicted high similarities with 4066 insects and micro-organisms. The sequence of salivary proteins like Actin related protein 1 isoform (4066), Elongation factor 1-alpha (1691), Tubulin beta chain (391), Heat shock protein 83 (449), and Heat shock protein 70 (386) had also shown close resemblance with the protein sequence of considerable insects and micro-organisms at a 50% similarity level. The biochemical activity and property of these salivary protein comprises like enzymes, binding proteins, putative effectors and regulatory proteins (mainly transcription factors). Overall, we observed a significant level of similarity across species, typically exceeding 50% identity between the rose-grain aphid and the rest of phloem-feeding insects and plant pathogens. This allowed us to anticipate conserved salivary proteins and contribute to a deeper comprehension of the molecular processes involved in the interaction between aphids and hosts.

The discovery of some salivary proteins in the liquid saliva of sap-sucking insects led to the exploration of homologous proteins through sequence comparisons among aphid species. The BLAST score ratio analysis was employed to visualize the extent of salivary protein similarity among different aphid species. The findings revealed that 46.67% (7) of the salivary proteins were conserved among the three or more aphid species (Table 4). Using the predicted salivary proteins of the rose-grain aphid as a query sequence, we have observed that 60% of the total salivary proteins exhibited close similarity with proteins of *Aphis craccivora*, while 46.67% of these proteins showed high similarity to those in *A. glycines*, *M. sacchari* and *S. flava*. Nevertheless, 40% of the salivary proteins were shared with *Aphis gossypii*. Meanwhile, only a few numbers of salivary proteins from the rose-grain aphid, 20% and 13.3%, displayed close similarity with *Rhopalosiphum padi* and *Myzus persicae*, respectively. 

In general, of the total salivary proteins discovered in the saliva of *M. dirhodum*, 60% had shown a close resemblance to aphids that primarily depend on pulse crops as a food source and sustenance (*Aphis craccivora*, *Aphis glycines*, *A. pisum* and *A. gossypii*). In contrast, only 46.67% of the salivary protein sequences of the rose-grain aphid displayed close similarities with cereal aphids, including *S. graminum*, *S. avenae*, *R. padi*, *M. persicae*, *Melanaphis sacchari* and the yellow sugar cane aphid, *Sipha flava* (Table 4). The close similarity in the biological makeup and composition of salivary proteins between rose-grain aphids and pulse crop aphids may indicate that they employ comparable functional and/or behavioral adaptation strategies, setting them apart from cereal aphids.

## 4. Discussion

In this research, we employed a multidisciplinary strategy to characterize the transcriptome sequence of rose-grain aphid, *M. dirhodum*, and to identify potential secretory proteins. The study of the rose-grain aphid’s head transcriptome and salivary proteome revealed four distinct trends: (1) a significant number of head transcriptome secretory proteins possess signal peptide sequences; (2) some portion of the head transcriptome proteins associated with secretory proteins have unknown functions; (3) with the exception of four proteins most saliva proteins had no secretion signals associated with each protein sequence; and (4) with the exception of four proteins, most secretory proteins predicted in the transcriptome of the head tissue could not be detected in the saliva of rose-grain aphid. This inconsistency is likely attributable to the highly specialized role of aphid saliva in adapting to the diverse and evolving defense mechanisms of plants.

Some of the inconsistencies between the salivary gland transcriptome and proteome might stem from the differences in expression levels between gene transcripts and proteins. Expression profiles of mRNA are highly dynamic, and there is often a lack of direct correlation between mRNA expression and protein levels [25]. Therefore, discrepancies between the expressed transcript and the resulting protein are likely to be common. Nonetheless, the goal of our study was to provide an initial catalogue of potential secreted proteins from the head tissue, utilizing both transcriptomic and proteomic data. The amalgamation of these datasets represents a crucial initial stride in pinpointing potential secretory proteins for forthcoming aphid studies.

### 4.1. Putative Effectors

At present, there is no functional data to clarify the roles of the aphid salivary proteins identified in this study. Nevertheless, possible effector functions for many of these candidate proteins can be inferred from their homology or similarity to effector proteins involved in pathogenesis or parasitism, as observed in other sap-sucking insects and plant pathogens. Host plants meticulously detect and respond to incoming injuries caused by the release of secretory proteins and mechanical damage from herbivorous insects. However, plant-feeding insects subsequently suppress immune responses triggered by these elicitors. Consistent with the finding of Huang, et al. [26], putative sheath proteins (encoded after the gene *shp-1*) were predicted in both saliva and the transcriptomic sequence of rose-grain aphid heads. According to Huang, et al. [26], the putative sheath protein (LsSP1) binds to the salivary sheath via the mucin-like protein (LsMLP1) when released into plants during feeding and probing. These proteins, which are particularly expressed in the head tissues of sap-sucking insects, are released into plants to suppress host defense mechanisms.

Secretory proteins, like glucose dehydrogenase protein-1 (secretory proteins encoded after the gene *gld-1*) and elongation factor 1 alpha (regarded as functional product of EF1A_0 gene) were also detected in the secretome and transcriptome of rose-grain aphid heads. The glucose dehydrogenase like protein 1 facilitates the oxidation-reduction process [27]. Ubiquitous plant defense responses against invading plant pathogens and insect pests may be degraded by proteins involved in oxidoreductase activity [28]. Thus, it is a potential effector that deters plant defense responses against aphid infestation. Glucose dehydrogenase like protein 1 also plays an indispensable role in insect development and the suppression of plant defense mechanisms.

Lipid-binding and transporting proteins (apolipophorins), (Cluster-3959.1281), were predicted in the transcripts of secretory genes. These proteins are homologous among some aphid species, like the potato aphid *Myzus euphorbiae* and *M. persicae*. The fecundity rate of *M. persicae* was increased following the overexpression of Me10 in *N. benthamiana*, indicating a species-specific ability to deter plant defense responses [29]. Beta-glucosidase transcript (Cluster-9628.0) were predicted from the transcriptome of the *M. dirhodum* head. It is a typical enzyme in termites (*Neotermes koshunensis*) that aids in degrading hemicellulose [30]. On top of this, it may also serve as a signaling pathway that triggers plant defense responses against herbivores. The treatment of cabbage leaves with beta-glucosidase induces the release of volatile compounds like salicylic acid (SA), ethylene and H_2_O_2_ [31].

Gene transcripts, specifically Cluster-3959.2598, Cluster-3959.726, Cluster-1501.0, Cluster-45.0, Cluster-671.0 and Cluster-4581.0 were predicted to encode for glucose dehydrogenase. By the same token, glucose dehydrogenase was also detected in the saliva of the rose-grain aphid (glucose dehydrogenase-like protein 1 and glucose dehydrogenase-like protein 2). These proteins were reported in the saliva and salivary glands of some aphid species, like *M. persicae* [5], *S. avenae* [28,32] and *A. craccivora* [33]. Glucose dehydrogenase is a potential effector that deters pattern triggered immune responses against aphid infestation.

The transcripts of lipase (Cluster-7728.1) and phospholipases (Cluster-3959.2585, Cluster-10963.0 and Cluster-7682.0) were extrapolated from the transcriptomic sequence of *M. dirhodum* heads with salivary glands. Lipase and phospholipase hydrolyze the linkages of triacylglycerols and phospholipids, respectively [34]. Unlike phospholipase, lipid processing, food digestion and transport have been enhanced by the activity of lipase [31]. In the transcription of secretory proteins (Cluster-3959.2585, Cluster-12716.1, Cluster-10963.0 and Cluster-7682.0), phospholipases are often involved in lipid biosynthesis and acyltransferase activity (namely catalysis of the transfer of an acyl group to an oxygen atom), calcium ion and receptor binding activities. The calcium binding activity of the phospholipase protein subsequently leads to the prevention of calcium-mediated occlusion and obliteration of phloem sieve elements against aphid feeding. Characterization of putative effectors of pea aphid using proteome mass spectrometry indicated both metalloprotease and calcium-binding proteins inactivate plant defense responses and inhibit the calcium-mediated blockade of plant sieve elements, respectively [8]. However, both lipase and phospholipase have versatile roles in various biological and industrial applications. The byproduct of phospholipase, phosphatidic acid, plays an important role in signal transduction cascades and lipid metabolic pathways, thus determining plant response to stress. When plants are exposed to stress such as mechanical stress (wounding and frost) and biological stress (pathogen and insect attack), the expression and activity of the phospholipase D protein increases rapidly [35]. Based on the findings of [36], rice infection with *Xanthomonas oryzae* increased phospholipase accumulation in its plasma membrane. Phospholipases also trigger plant defense responses (the release of abscisic acid, ethylene and nicotinamide adenine dinucleotide phosphate ion (NADPH)) against herbivorous insects and diseases [35]. The transcript of trehalose (Cluster-608.0) facilitates the expression of trehalose transporter Tret1-2 homolog isoform X2, trehalose-phosphatase, and alpha-trehalose-phosphate synthase. In plants, trehalose plays a regulatory role in sugar metabolism, growth, development, and responses to stress induced by biotic and abiotic factors [37]. An overexpression of the trehalose biosynthetic gene causes the plant and microbes to receive exogenous trehalose, thereby increasing tolerance to stress [38,39]. Trehalose has also been reported in the salivary glands of *B. tabaci* and *S. avenae* [40,41]. Based on the findings of [41], trehalose plays an important role in deterring the accumulation of excess trehalose in plant cells to suppress immune responses.

### 4.2. Detoxifying Secretory Proteins

When plants are attacked by herbivorous insects, they release secondary toxic metabolic products (mainly protease inhibitors). Nonetheless, herbivorous insects secrete detoxifying proteins to degrade the toxins produced upon feeding, thus paving the way for adaptation to adverse conditions. Esterase is one of the detoxifying proteins released by aphids to manipulate a plant’s secondary metabolites and insecticides [42]. The transcripts of secretory genes (Cluster-6992.0 and Cluster-6189.0) were predicted to encode the secretory protein, esterase E4. This protein breaks down the effect of chemical pesticides when farmers sprayed their crops to manage aphids. A prevalent strategy utilized by *Myzus persicae* aphid to build resistance to insecticides involves boosting the expression of genes that encode for esterase E4 [43].

Secretory proteins such as glutathione S-transferase 6 and CAAX prenyl protease 1 homolog (ortholog metalloproteases) were also predicted in the transcriptome assembly of the head tissue after gene transcripts of Cluster-11681.0 and Cluster-3959.1466. Glutathione S-transferases allow insects to survive under chemical stress through the metabolism of xenobiotics or by providing protection against oxidative stress [44]. Although there is insufficient evidence to support our findings, a report from [45] indicated that many types of metalloproteinases are involved in detoxifying the secondary metabolites of plants.

Clusters of gene transcripts (Cluster-7473.0, Cluster-477.0, Cluster-3959.111, Cluster-3959.1431, Cluster-3959.824, and Cluster-3959.2743) were also predicted encoding secretory proteins, specifically peroxidase and oxidoreductase. These proteins are essential for basic biological functions, such as responses to stress and defense mechanisms. Previously, the secretory proteins were discovered in some aphid species, mainly *in S. avenae* [41], *A. pisum* [8], *Mayetiola destructor* [46] and *Megoura viciae* [45]. Unlike other species of aphids, only superoxide dismutase (Cluster-3959.779) was predicted in the transcriptomic sequences of *M. dirhodum*. Research conducted on parasitoid wasp (*Leptopilina heterotoma*), revealed that extracellular superoxidase dismutase was synthesized and released along with the fluid and acts as virulence factors that counteract the immune response of *Drosophila species* [47]. It is also used by pathogenic bacteria, fungi and pathogenic protozoan parasites as virulence factors against the resistant gene of the host during early infection. This protein initiates plant defense responses when aphids begin to probe and feed on plants. Thus, it is considered to be a signaling pathway in the plant defense system. Peroxidase transcripts (Cluster-3959.111, Cluster-3959.824 and Cluster-7473.0) were predicted in transcriptomic sequences of secretory proteins from *M. dirhodum* aphids. Based on findings from [41], the presence of peroxidase in aphid saliva serves as an antioxidant involved in the scavenging of hydrogen peroxides and thus plays a significant role as a detoxifying enzyme and in suppressing reactive oxygen species-induced plant defense responses.

### 4.3. Digestive Secretory Proteins

The probing and feeding activities of aphids are also enhanced by digestive enzyme release into the host. The transcript of serine protease (Cluster-12396.0) was assembled from the transcriptomic sequences of rose-grain aphids. Serine protease is a digestive enzyme secreted by the midguts of phloem-feeding insects to facilitate the digestion of food assimilates while ingesting phloem saps from the plant [48]. Serine protease also acts against host-related serine protease inhibitors and phenol oxidase defense response. Secretory gene transcripts, namely Cluster-1194.0, Cluster-1892.0, Cluster-2182.0, Cluster-2350.0, Cluster-3666.0, Cluster-3959.1850, Cluster-3959.2179, Cluster-3959.964, Cluster-6237.0 and Cluster-7233.0 were assembled and predicted to encode for digestive secretory proteins, most likely trypsin. Trypsin is secreted by phloem-feeding aphids to facilitate the break down and oral digestion of plant phloem constituents [42].

### 4.4. Ca^2+^ Binding Secretory Proteins

The putative calcium-binding protein, regucalcin, was predicted in the transcriptional product of the secretory gene, Cluster-11867.0, assembled from the transcriptomic sequence of *M. dirhodum* heads with salivary glands. Similarly, in this experiment we had predicted sarcalumenin (SAR) from the secretory gene transcript, Cluster-3959.1170. Sarcalumenin is associated with calumenin calcium buffer proteins to modulate the uptake of calcium ions and release the excitation and contraction of muscle fibers. It also plays an important role in other physiological functions, such as muscle resistance against fatigue, muscle development, sarco-endoplasmic reticulum calcium ATPase stabilization, and store-operated calcium entry mechanisms [49]. Plants release calcium ions into the sieve element lumen to block the outflow of phloem saps during mechanical damage, feeding, and probing. In response, sap-sucking insects release water saliva consisting of calcium ion-binding proteins into the lumen of the sieve element to prevent the plugging and clogging of feeding sites for insects [50]. With the aid of transcriptomic and proteomic approaches, this protein was detected in the saliva and salivary glands of some homopterous insects such as *A. pisum* and *Nephotettix cincticeps*. These results revealed that the synthesis and secretion of calcium-binding proteins could be a means of suppressing plant defense responses against all phloem sap-sucking insects [51].

### 4.5. Zn-Binding Secretory Proteins

The transcripts of aminopeptidase N (Cluster-8442.0, Cluster-11978.0 and Cluster-7461.0) were detected in the transcriptomic sequences of *M. dirhodum* secretory proteins. This protein is implicated in binding zinc ions and also known for metallopeptidase activity during aphid-host interactions. Generally, Aminopeptidase N is a multifunctional protein and expressed in different organs and cells of many insects [52]. This protein catalyzes the breakdown of the amide group of amino acids from the N terminal of proteins or peptides. Other versatile types of aminopeptidases have also been identified in *M. dirhodum* aphid transcripts. Endoplasmic reticulum aminopeptidase 2 was predicted to be synthesized from the transcripts of secretory proteins (Cluster-3959.627) and was found to perform important molecular functions, such as metallopeptidase, zinc binding, and hydrolysis. The protein acts against ester bonds of the amino acid, thereby facilitating the disintegration of the disulfide bonds of polypeptides and/or proteins. The functional product of the transcript of gene (Cluster-3959.1656), that is, the thyrotropin-releasing hormone-degrading ectoenzyme, is also an integral part of the aminopeptidase protein typically secreted in the salivary glands of insects and functions as the binding of zinc ions (to the active site of the protein, aminopeptidase) and other metallopeptidase activities (penetration of plant cell wall and membrane while probing their stylet to the layers of a cell’s phloem sieve element) at a specific amino acid location of the protein. Clusters of unigenes transcripts, Cluster-3959.1011, Cluster-3959.1012 and Cluster-7461.0 were assembled from the transcriptomic sequences of aphid heads and predicted to encode metallopeptidase and lysosomal alpha-mannosidase proteins, which in turn bind zinc. Zinc, a crucial micronutrient vital for the growth, development and defense of all living things, plays a significant role in the interaction between hosts and pests in plant systems, where competition for Zn can influence the outcome [53].

### 4.6. Reproduction and Development

Vitellogenin (encoded after gene *vit-6*) was detected in the proteome and transcriptome (Cluster-1741.0) of *M. dirhodum* head tissue. It is synthesized in the fat body, and plays multiple roles in insect reproduction, mainly during oocytes and embryo development. Vitellogenin receptors (VgR) present on the surface of oocytes, are responsible for vitellogenin transportation from haemolymph to oocytes [54]. The vitellogenin genes have been extensively investigated in root-knot nematodes, *Caenorhabditis elegans* [55]. Clusters of secretory gene transcripts, Cluster-3959.2780 and Cluster-4827.0, were also predicted to encode for secretory proteins (protein slit). This secretory protein plays an essential role as a midline repellent, stopping longitudinal axons from crossing the central nervous system’s midline in a range of insects, nematodes and planarians [56].

### 4.7. Protein Synthesis and Secretion

Disulfide isomerase was identified from the transcriptome of head tissues, but it was not notably detected in the proteomic profile of salivary proteins. Protein disulfide isomerases are widespread and versatile enzymes found in eukaryotic organisms. These enzymes belong to the thioredoxin superfamily. Classical disulfide isomerases are highly conserved and include an N-terminal signal peptide [57]. This enzyme is believed to play a role in protein folding regulation. Additionally, disulfide isomerase was found in the salivary secretions of plant-parasitic nematodes and has been associated to production of salivary proteins [58,59].

### 4.8. Odorant Binding and Chemosensory Proteins

In addition to visual signals, aphids largely depend on certain metabolic products as cues to identify their host within complex and spatially diverse environments [60]. In line with the information of Shih, et alet al. [60], a chemosensory protein was predicted among the secretary genes (Cluster-3959.2546) assembled from the transcriptomic sequence of *M. dirhodum* aphids. Previously, chemosensory proteins were reported in the transcriptome of salivary glands from *N. lugens*, *M. persicae*, *M. cerasi*, and the bird cherry-oat aphid *R. padi* [61]. According to a review by Shih, et alet al. [60], multiple gene products typically chemosensory proteins, odorant binding proteins, gustatory receptors, and olfactory receptors are important in locating and exploiting host resources following the release of phenolic compounds from the plant. In insects, the transportation of organic compounds that convey specific chemical signals (semio-chemicals) is performed with the help of chemosensory proteins. Aphids communicate with each other and locate their food sources and ovipository sites with the aid of odorant binding and chemosensory proteins expressed and released through their antennae and mouth [62]. However, odorant binding and chemosensory proteins may also be located in other parts of insect tissues or organs, mainly the legs, head, thorax, abdomen, and salivary glands from the head. The function of these proteins may differ depending on the location of their expression, whereby they may aid in insect development, the regeneration of insect legs, host interactions and immune responses [63,64]. The product of unigene transcripts (Cluster-3959.2546), i.e., chemosensory proteins, predicted from the transcriptome sequence of *M. dirhodum* showed high similarity to proteins reported in *S. avenae* and *M. persicae* aphid species. According to a functional assay carried out on *Nicotiana benthamiana*, the overexpression of Mp10 protein in *N. benthamiana* suppressed bacteria-associated molecular patterns and local cell death that impede the fecundity of *M. persicae* [65]. The odorant binding and chemosensory proteins of *Spodoptera frugiperda* recognize chemical, behavioral and/or physiological responses of plants [66].

## 5. Conclusions and Recommendation

The successful colonization of plants by invading pathogens hinges on a variety of secretory proteins released into the host to either suppress or modulate specific innate defenses. This study aimed to determine whether the rose-grain aphid possesses a similar array of potential secretory proteins as other phloem-feeding insects. Our analysis of the rose-grain aphid head tissue secretome revealed three key characteristics: (1) a significant portion of head tissue proteins possess secretion signal (signal peptides); (2) four salivary proteins identified in the saliva of rose grain aphids consisted of signal peptides associated with the N-terminal of amino-acid sequences; and (3) there have been notable similarities in secretory protein compositions between rose-grain aphid head tissue and other aphid species. A transcriptomic analysis of *M. dirhodum* aphids revealed 18,030 genes from 31,344 transcripts in the head tissue along with salivary glands, with 705 genes encoding secretory proteins. Notably, 28.5% of the total gene transcripts were deemed to have unknown functions in the Nr database. The proteomic analysis unveiled the discovery of 15 salivary proteins, including enzymes, binding proteins, putative effectors and regulatory proteins, highlighting their role in aphid-plant interactions. Despite lacking signal peptides, salivary proteins such as Actin related protein 1 isoform, Elongation factor 1-alpha, Tubulin beta chain, Heat shock protein 83, ATP synthase subunit alpha, Histone H2B and one hypothetical protein were found in the watery saliva, suggesting unknown secretion mechanisms. A total of 46.67% of these proteins are conserved across three or more aphid species. Furthermore, 60% of the total salivary protein showed close similarity to *Aphis craccivora* and 46.67% of these proteins had close similarity with *A. glycines*, *M. sacchari* and *S. flava*. Nevertheless, 40% of the salivary proteins were shared with *Aphis gossypii*. However, only 20% and 13.3% of these proteins were similar to those of *Rhopalosiphum padi* and *Myzus persicae* respectively. A total of 60% of the salivary proteins of rose-grain aphids closely matched to aphids targeting leguminous crops. Hence, rose grain aphid may share functional and/or behavioral adaptive strategies with pulse aphids. This study underscores the need for further research on gene expression profiling and functional analyses of salivary proteins to elucidate their nature, expression and role in aphid-plant interactions.

## Figures and Tables

**Table 1:** Quality and assembly of the transcriptome sequence of head tissues.

S/N	Statistic	Read
1	Raw Reads (bp)	47,565,328
2	Clean Reads (bp)	46,238,772
3	Clean Bases (Gb)	6.94 Gb
4	Error (%)	0.03
5	Q_20_ (%)	97.61
6	Q_30_ (%)	93.04
7	N percentage (%)	0
8	Total length of transcripts	48,023,560
9	Total length of genes	25,480,502
10	Number of Transcripts	31,344
11	Number of genes	18,030
12	Mean Length of Transcript	1532
13	Mean Length of gene	1413
14	N50 Transcript	2335
15	N50 Genes	2205
16	GC Content (%)	42.47

**Table 2:** Number and percentage of gene transcripts annotated against all known data bases.

S/N	Data Base	Number of Genes	Percentage of Genes (%)
1	Nr	12,589	69.82
2	Nt	14,253	79.05
3	KO	5587	30.98
4	Swiss-Prot	9467	52.5
5	Pfam	9314	51.65
6	GO	9314	51.65
7	KOG	5850	32.44
8	All data bases	3670	20.35
9	At least one data base	14,887	82.56
10	Total Genes	18,030	100

**Table 3:** Sequence similarity of secretory (comprising signal peptide) and non-secretory (lacking signal peptide) salivary proteins among different organisms at three similarity level.

Proteins Identified in the Saliva of *M. dirhodum*	Entry	Secretory Nature		Similarity Level		
		Yes	No	100%	90%	50%
Actin related protein 1 isoform X1	A0A6G0YHT2		√	182	190	4066
ATP synthase subunit alpha, mitochondrial	A0A6G0YU19		√	1	1	10
Elongation factor 1-alpha	A0A2H8TTF1		√	13	373	1691
Glucose dehydrogenase like-protein 1	K0DCK7		√	x	2	2
Glucose dehydrogenase like-protein 2	K0D9J0	√		x	x	2
Heat shock protein 83	A0A8R2AZJ4		√	1	14	449
Heat shock protein 70KD	A0A5E4MRQ3	√	√	2	16	386
Histone H2B	A0A2S2N8A9		√	1	1	175
Histone H4 (Fragment)	A0A6G0XR79		√	30	30	32
Peroxiredoxin 1	A0A2S2NYR8		√	1	1	17
Putative sheath protein (Fragment)	K0D9J4	√		x	x	2
Tubulin beta chain	A0A6G0ZHT7		√	6	53	133
Uncharacterized protein	A0A8R2NN35		√	1	8	14
Vitellogenin domain-containing protein	A0A8R2F8V9	√		1	1	9
HSC70b (Fragment)	V5KS27		√	1	1	1

**Table 4:** Sequence similarity of salivary proteins among different aphid species based on BLAST score ratio (BSR).

Protein Names Identified from the Saliva of *M. dirhodum*	Accession Number	Pulse Crop Aphids				Cereal Crop Aphids					
		*A. craccivora*	*A. glycines*	*A. pisum*	*A. gossypii*	*S. graminum*	*S. avenae*	*R. padi*	*M. persicae*	*M. sacchari*	*Sipha flava*
Actin related protein 1 isoform X1	A0A6G0YHT2	√	√	√	√	x	√	√	x	√	√
ATP synthase subunit alpha, mitochondrial	A0A6G0YU19	√	x	x	x	x	x	x	x	x	x
Elongation factor 1-alpha	A0A2H8TTF1	x	x	x	x	√	x	x	x	√	x
Glucose dehydrogenase like-protein 1	K0DCK7	x	x	x	x	x	√	x	x	x	x
Glucose dehydrogenase like-protein 2	K0D9J0	x	x	x	x	x	√	x	x	x	x
Heat shock protein 83	A0A8R2AZJ4	√	√	√	√	x	x	√	√	√	√
Heat shock protein 70KD	A0A5E4MRQ3	√	√	√	√	x	x	√	√	√	√
Histone H2B	A0A2S2N8A9	√	√	x	x	√	x	x	x	x	x
Histone H4 (Fragment)	A0A6G0XR79	√	x	x	x	x	x	x	x	x	x
Peroxiredoxin 1	A0A2S2NYR8	x	x	x	x	√	x	x	x	x	√
Putative sheath protein	K0D9J4	x	x	x	x	x	√	x	x	x	x
Tubulin beta chain	A0A6G0ZHT7	√	√	x	√	x	x	x	x	√	√
Uncharacterized protein	A0A8R2NN35	√	√	√	√	√	x	x	x	√	√
Vitellogenin domain-containing protein	A0A8R2F8V9	√	√	√	√	x	x	x	x	√	√
HSC70b (Fragment)	V5KS27	x	x	x	x	x	√	x	x	x	x
		9	7	5	6	4	5	3	2	7	7

## Data Availability

These materials are made available free of charge via the Internet portal at https://zenodo.org/uploads/11634547 (accessed on 5 February 2024). Meanwhile, the transcriptome sequence data have been deposited in the Sequence Read Archive (SRA) of the National Center for Biotechnology Information (NCBI) database with the accession number PRJNA1134911 (http://www.ncbi.nlm.nih.gov/bioproject/ PRJNA1134911, accessed on 5 February 2024).

## Supplementary Materials

The following supporting information can be downloaded at: https://www.mdpi.com/article/10.3390/cimb46120798/s1, Supplementary files, List of unigenes assembled from the transcriptomic sequence of rose grain aphid (Table S1) and List of secretory genes and gene products predicted from total unigenes (Table S2). Supplementary file of salivary proteins of Rose Grain Aphid, List of salivary proteins identified in the saliva of Metopolophium dirhodum by Mascot software (Result one) and List of salivary proteins identified in the saliva of Metopolophium dirhodum by MaxQuant software (Result two).
